# Neuregulin-1 inhibits CoCl_2_-induced upregulation of excitatory amino acid carrier 1 expression and oxidative stress in SH-SY5Y cells and the hippocampus of mice

**DOI:** 10.1186/s13041-020-00686-2

**Published:** 2020-11-13

**Authors:** Han-Byeol Kim, Ji-Young Yoo, Seung-Yeon Yoo, Jun-Ho Lee, Wonseok Chang, Hye-Sun Kim, Tai-Kyoung Baik, Ran-Sook Woo

**Affiliations:** 1 Department of Anatomy and Neuroscience, College of Medicine, Eulji University, 143-5Jung-Gu, Yongdu-Dong, Daejeon, 301-746 Republic of Korea; 2 Department of Emergency Medical Technology, Daejeon University, Daejeon, 34520 Republic of Korea; 3 Department of Physiology, College of Medicine, Eulji University, Daejeon, 301-746 Republic of Korea; 4 Department of Pharmacology, College of Medicine, Seoul National University, Seoul, 110-799 Korea; 5 Seoul National University College of Medicine, Bundang Hospital, Sungnam, 13620 Republic of Korea

**Keywords:** CoCl_2_, EAAC1, Neuregulin-1, Oxidative stress, Apoptosis

## Abstract

Excitatory amino acid carrier 1 (EAAC1) is an important subtype of excitatory amino acid transporters (EAATs) and is the route for neuronal cysteine uptake. CoCl_2_ is not only a hypoxia-mimetic reagent but also an oxidative stress inducer. Here, we found that CoCl_2_ induced significant EAAC1 overexpression in SH-SY5Y cells and the hippocampus of mice. Transient transfection of EAAC1 reduced CoCl_2_-induced cytotoxicity in SH-SY5Y cells. Based on this result, upregulation of EAAC1 expression by CoCl_2_ is thought to represent a compensatory response against oxidative stress in an acute hypoxic state. We further demonstrated that pretreatment with Neuregulin-1 (NRG1) rescued CoCl_2_-induced upregulation of EAAC1 and tau expression. NRG1 plays a protective role in the CoCl_2_-induced accumulation of reactive oxygen species (ROS) and reduction in antioxidative enzyme (SOD and GPx) activity. Moreover, NRG1 attenuated CoCl_2_-induced apoptosis and cell death. NRG1 inhibited the CoCl_2_-induced release of cleaved caspase-3 and reduction in Bcl-X_L_ levels. Our novel finding suggests that NRG1 may play a protective role in hypoxia through the inhibition of oxidative stress and thereby maintain normal EAAC1 expression levels.

## Introduction

Excitatory amino acid carrier 1 (EAAC1, also referred to as EAAT3) is one neuronal subtype of excitatory amino acid transporter (EAAT) that is ubiquitously expressed in the central nervous system (CNS). EAAC1 can also transport cysteine at a rate comparable to that of glutamate and is the primary route for the uptake of neuronal cysteine. Cysteine is a critically important substrate for the synthesis of glutathione (GSH), one of the most important intracellular antioxidants in the brain [1, 2]. Mature neurons utilize cysteine but not cystine for GSH synthesis [3, 4]. EAAC1-mediated uptake may be the major source of cysteine for GSH synthesis in mature neurons [5]. Oxidative stress is a general premonitory hallmark of numerous brain pathologies and largely contributes to the acute and chronic outcomes of CNS disorders, such as epilepsy, ischemic stroke, amyotrophic lateral sclerosis, Alzheimer’s disease and Parkinson’s disease [6]. Modulation of EAAC1 activity correlates with neuronal GSH levels [7]. Knockdown of EAAC1 reduces cysteine uptake and intracellular GSH levels [8].

The intracellular response to hypoxia is regulated by hypoxia inducible factor-1 (HIF-1). HIF-1 is a transcription factor, and a heterodimer consisting of an oxygen-dependent regulatory HIF-1α subunit and a constitutively expressed HIF-lβ subunit that acts as a master regulator of adaptation to a low oxygen environment in the cell [9]. Recent evidence suggests that the ROS produced in the mitochondria mediate HIF-1α stabilization during hypoxia [9]. Hypoxia leads to a rapid increase in spontaneous vesicular glutamate release [10] and impaired glutamate uptake [11–13]. EAAC1 was increased at the transcript level in C6 cells by hypoxia [14]. Oxygen–glucose deprivation (OGD) induced the protein expression of EAAC1 in pure and mixed neuronal cultures and promoted EAAT3 activity, which increased glutamate uptake into cultured neurons [15]. EAAC1 transcript levels were transiently upregulated during the reperfusion phase in ischemia–reperfusion models [15]. Ischemia–reperfusion leads to oxidative stress and an accompanying transient increase in EAAT3 immunoreactivity in the hippocampus [16].

Neuregulin-1 (NRG1) is a member of the NRG family of growth factors that play important roles in the developing and adult CNS [17]. Recently, accumulating evidence has collectively shown that NRG1 is a new regulator of injury and repair with multifaceted roles in neuroprotection, remyelination, and immunomodulation. NRG1 protects against a number of CNS pathological conditions, including ischemia, neurotrauma, and neurodegenerative diseases [18–21, 23]. Our recent work showed that NRG1 regulated hypoxia-inducible factors such as HIF-1α and p53 [24]. NRG1/ErbB4 attenuates neuronal cell damage under OGD in primary hippocampal neurons [25]. These findings suggest a correlation between NRG1 dysfunction and CNS pathology. Therefore, NRG1 may be a potential therapeutic target in the recovery of function after CNS injury.

Herein, we used cobalt chloride (CoCl_2_), a hypoxia mimic, to induce oxidative stress in SH-SY5Y cells. Cobalt stimulates reactive oxygen species (ROS) generation through a nonenzymatic, nonmitochondrial mechanism, and CoCl_2_ treatment induces HIF-1α accumulation [26].

Our study provides conclusive molecular evidence that CoCl_2_ strongly induces EAAC1 expression in SH-SY5Y cells and hippocampus of mice. These acute changes may response against of reactive oxidative stress. NRG1 reduced the CoCl_2_-induced oxidative and thereby rescue upregulation of EAAC1.

## Materials and methods

### Reagents and antibodies

Recombinant β-type NRG1 was purchased from ProSpec (East Brunswick, NJ, USA). Antibodies were obtained from Millipore Corporation (Chemicon, MA, USA) (EAAT3 (EAAC1), MAB1587), Santa Cruz Biotechnology Inc. (Santa Cruz, CA, USA) (Bcl-X_L_, sc-8392; p53, sc-126; β-actin, sc-47778), Novus Biologicals (Centennial, CO, USA) (HIF-1α, NB100-131; Tau, NBP-25613), ThermoFisher scientific (Waltham, MA, USA) (Phospho-Tau (AT8), #MN1020), Mybiosoure (San Diego, CA, USA) (Phospho-Tau (ser422), #A11008), and Cell Signaling Technology (CST, MA, USA) (Caspase 3, #9662 s; cleaved caspase 3, # 9661 s; EAAC1, #12,179; Myc-tag, #2276; HRP-conjugated anti-rabbit IgG, #7076 s; HRP-conjugated anti-mouse IgG, #7074 s). CoCl_2_ (C8661) was purchased from Sigma-Aldrich (St. Louis, MO, USA).

### Animals and stereotaxic surgery

C57BL/6 (male, 10 weeks old, 24–27 g) mice were obtained from a laboratory animal supplier (Samtako Bio Korea) and were housed in cages under standard laboratory conditions with a 12-h light/12-h dark cycle. A total of twenty animals were randomly allocated to the following four groups: saline (n = 8), NRG1 (n = 8), CoCl_2_ (n = 8), and CoCl_2_ + NRG1 (n = 8). Experiments with animals were approved by the Institutional Animal Care and Use Committee of Eulji University (EUIACUC 19–08). All surgical procedures and perfusions were performed under anaesthesia via intraparietal injection of ketamine (100 mg/kg) with Rompun (10 mg/kg). The animals were subjected to a unilateral lesion by placing them in a stereotaxic apparatus. CoCl_2_ (25 mM) was delivered in the ventral hippocampus of the right hemisphere (coordinates from bregma: anterior/posterior − 3.3 mm, medial/lateral + 2.8 mm, dorsal/ventral − 4.0 mm). Each microinjection unit was attached to a 10-μl Hamilton microlitre syringe via a glass tube, and administration was controlled by the experimenter at a rate of 1 μl (volume injected) over a period of approximately 2 min 30 s.

### Cell culture and transfection

SH-SY5Y human neuroblastoma cells were purchased from the American Type Culture Collection (Manassas, VA, USA) and cultured in Dulbecco’s modified Eagle’s medium (Invitrogen, Carlsbad, CA, USA) containing 10% fetal bovine serum (FBS) and a penicillin–streptomycin-amphotericin B mixture (Invitrogen) at 37 °C in a humidified atmosphere containing 5% CO_2_. When the cells grew sufficiently in 100 mm culture dishes (SPL Life Sciences, Gyeonggi-do, Korea), they were subcultured in 6-well or 96-well plates. SH-SY5Y cells were transiently transfected with either 4 μg of plasmid pcDNA3.1 (Mock) or pcDNA3.1-EAAC1-myc and 10 μl of Lipofectamine 2000 (Invitrogen) in 250 μl of Opti-MEM without serum according to the manufacturer’s instructions. Transient transfection efficiencies were confirmed by Western blot in SH-SY5Y cells.

### Assessment of cell death

Cell death after CoCl_2_ treatment was assessed by determining the release of lactate dehydrogenase (LDH) into the culture medium, thereby indicating a loss of membrane integrity. LDH activity was measured using a commercial kit (Cytotox 96 nonradioactive cytotoxicity assay kit, Promega, Madison, WI, USA) according to the manufacturer’s protocol. The absorbance was measured at 490 nm using a VICTOR X3 multilabel plate reader (PerkinElmer, Shelton, USA).

### TUNEL staining

In situ DNA fragmentation was assessed using a terminal deoxynucleotidyl transferase (TdT) dUTP nick end labeling (TUNEL) staining kit (Roche Diagnostics) according to the manufacturer’s instructions. Images were captured after counterstaining with 10 μM 4′,6-diamidino-2-phenylindole (DAPI; Invitrogen) for 30 min. The number of apoptotic cells was counted in five random fields using a Zeiss LSM 5 LIVE confocal microscope (Carl Zeiss AG, Oberkochen, Germany). The apoptotic cells are expressed as the percentage of TUNEL-positive cells in the total number of DAPI-stained cells.

### ROS measurement

ROS generation in SH-SY5Y cells was analyzed using the dye 2′,7′-dichlorodihydrofluorescein diacetate (DCFH-DA; Invitrogen, CA, USA). SH-SY5Y cells were washed three times with Dulbecco’s phosphate-buffered saline (DPBS) and then incubated at 37 °C in DPBS containing 20 μM DCFH-DA for 30 min. Once inside the cells, DCFH-DA is hydrolyzed by esterase to form polar DCFH, which then interacts with ROS. Cells were subsequently washed three times with DPBS and visualized with a fluorescence microscope (EVOS M5000, Thermo Fisher Scientific, Eugene, OR, USA) at an excitation wavelength of 485 nm.

### Glutathione peroxidase (GPx) activity assay

GPx activity was determined using a Biovision glutathione peroxidase activity assay kit (Cayman Chemical Company, MI, USA) according to the manufacturer’s protocol. SH-SY5Y cells were homogenized on ice in cold assay buffer and then centrifuged at 10,000×*g* for 15 min at 4 °C. Then, 50 μl of cell supernatant was added to a 96-well plate with 50 μl of assay buffer. The reaction mixture was added to each sample and incubated for 15 min to deplete all GSSG in the samples. Ten microliters of cumene hydroperoxide substrate was subsequently added to initiate the enzymatic reaction. The absorbance was immediately measured at a wavelength of 340 nm using a VICTOR X3 multilabel plate reader (PerkinElmer, Shelton, USA). GPx activity was calculated using an NADPH standard curve.

### Superoxide dismutase (SOD) activity assay

SOD activity was measured using a commercially available kit (Cayman Chemical Company, MI, USA) according to the manufacturer’s protocol. SH-SY5Y cells were homogenized in cold 20 mM HEPES buffer (pH 7.2) and centrifuged at 1,500×*g* for 5 min at 4 °C. Each sample (10 μl) was added to a 96-well plate with 200 μl of the diluted radical detector. Then, 20 μl of diluted xanthine oxidase was added to initiate the enzymatic reaction. The absorbance was immediately measured at a wavelength of 450 nm using a VICTOR X3 multilabel plate reader (PerkinElmer, Shelton, USA).

### Immunofluorescence analysis

SH-SY5Y cells were fixed using 4% paraformaldehyde and 4% sucrose in DPBS (pH 7.4) for 20 min at room temperature (RT). Next, the cells were permeabilized and blocked using DPBS containing 1% BSA and 0.1% Triton X-100 at RT for 30 min, and then primary antibodies (mouse anti-EAAC1 (1:100) and rabbit anti-tau (1:100)) were added and incubated overnight at 4 °C. The cells were then washed three times in PBS and incubated with Alexa Fluor 488 goat anti-mouse IgG and Alexa Fluor 594 goat anti-chicken IgG (Jackson ImmunoResearch Laboratories, Inc., 1:200) for 2 h at RT. After counterstaining with DAPI (10 μM in DPBS), the cells were mounted in Vectorshield (Vector Laboratories). Fluorescent images were acquired with an LSM 5 LIVE confocal system (Carl Zeiss AG, Oberkochen, Germany).

### Dihydroethidium (DHE) staining

To assess superoxide production, the brain was immediately frozen in embedding medium [22]. Briefly, post-fixed cryosections (15 µm) were incubated in DPBS containing 10 μM DHE (Invitrogen CA, USA) for 30 min at 37 °C in the dark room. The sections were then washed thrice with DPBS and mounted in Vectorshield (Vector Laboratories). Fluorescent images were acquired with an LSM 5 LIVE confocal system (Carl Zeiss AG, Oberkochen, Germany). Images were obtained using an excitation wavelength of 561 nm and an emission wavelength of 640 nm.

### Western blot analysis

Western blotting was performed as previously described [23]. Briefly, tissues were homogenized using a modified homogenization buffer (50 mM Tris–HCl [pH 7.4], 150 mM NaCl, 1% NP-40, 0.25% sodium-deoxycholate, 1 mM PMSF, 1 mM EDTA, and 1 μg/ml each of aprotinin, leupeptin, and pepstatin protease inhibitors). Samples were then resolved using SDS-PAGE, transferred to nitrocellulose membranes and subsequently blocked with TBS containing 5% fat-free milk and 0.05% Tween-20 for 1 h. Next, the membranes were incubated overnight at 4 °C with primary antibodies (anti-EAAC1, 1:1,000, Millipore Corporation; anti-cleaved caspase-3, 1:1,000, anti-caspase 3, 1:1,000, anti-Myc-tag, 1:1,000, Cell Signaling; anti-HIF-1α, 1:1,000, Novus Biologicals; anti-p53, 1:1,000, anti-β-actin, 1:5,000, Santa Cruz Biotechnology) and developed using horseradish peroxidase-conjugated secondary antibodies. Immunodetection was performed with a chemiluminescence system (Amersham Pharmacia) and a ChemiDoc TM tough imaging system (Bio-Rad, California, USA).

### Statistical analysis

The data are presented as the means ± SEM of three or more independent experiments. Student’s paired *t*-test was used for comparisons of the means between two groups of cells in a single experiment. For the data of more than two groups, statistical analyses were performed by one-way analysis of variance (ANOVA) followed by Bonferroni’s post hoc test. A value of *P* < 0.05 was considered statistically significant.

## Results

### CoCl_2_ increased EAAC1 protein expression in SH-SY5Y cells and the ventral hippocampus (VH) of mice

**Fig. 1:**
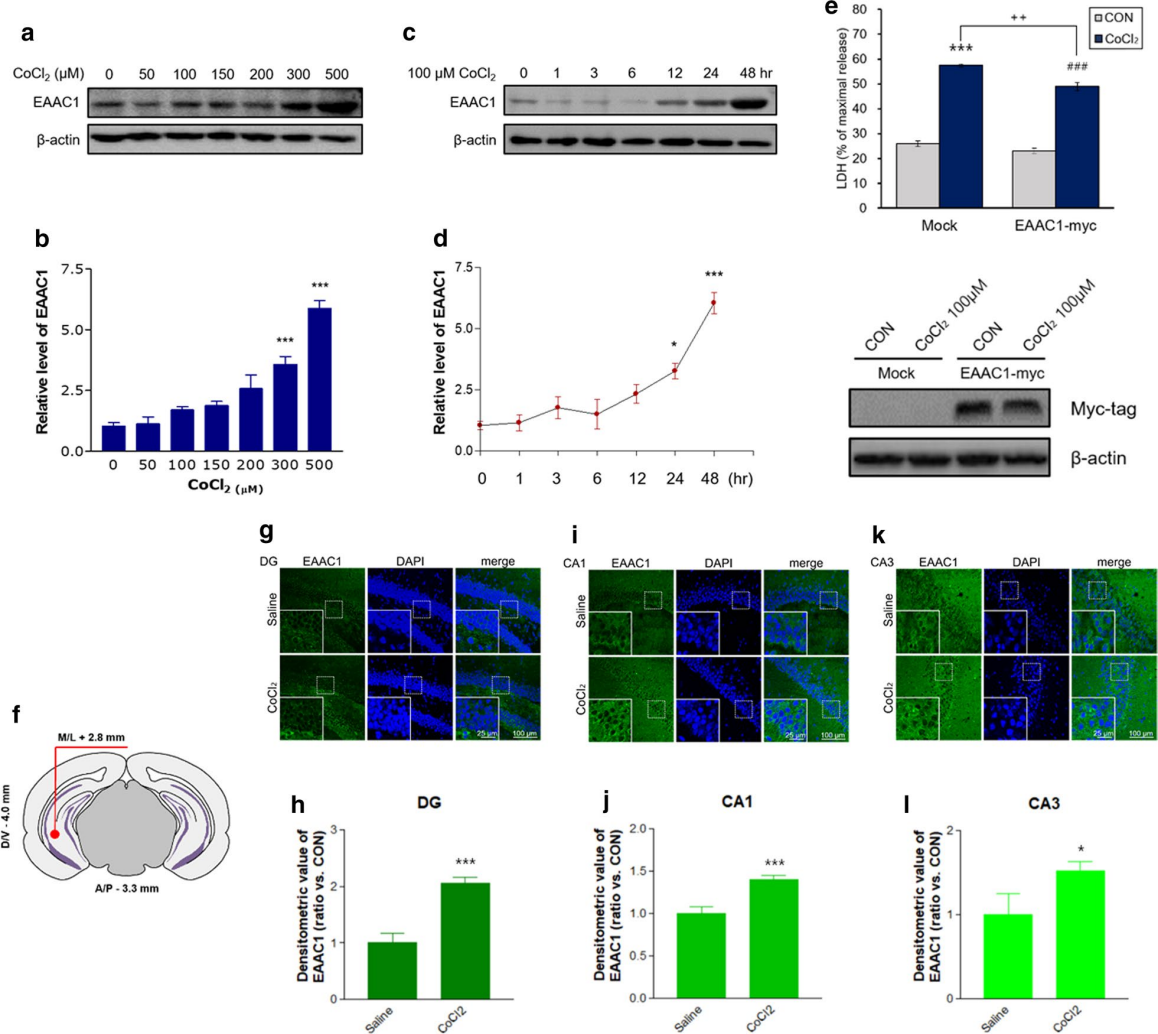
The expression of EAAC1 in CoCl_2_-treated SH-SY5Y cells. **a** SH-SY5Y cells were treated with different concentrations of CoCl_2_ (0, 50, 100, 150, 200, 300 and 500 µM) for 24 h, which resulted in dose-dependent increases in EAAC1 expression. **b** Quantitative analysis of EAAC1 immunoreactivity in **a**. The results are presented as the means ± S.E.M.; n = 8; ***P < 0.001. **c** Western blotting demonstrated that 100 µM CoCl_2_ affected EAAC1 protein expression levels. EAAC1 protein expression was significantly increased by 100 µM CoCl_2_ in a time-dependent manner. **d** Quantification of the data in c. The densitometry values are shown as ratios relative to the values of the control group (n = 6; *P < 0.05, ***P < 0.001). **e** The degree of cell death was assessed 36 h after EAAC1-myc transfection using LDH activity in the medium (Mock: n = 6; ***P < 0.001 versus the control group; EAAC1-myc: n = 6; ^###^*P* < 0.001 versus the control group; CoCl_2_ group: ^++^*P* < 0.01 versus the Mock group). Representative immunoblots for Myc-tag are shown for transfection with EAAC1-myc. **f** Illustration of procedure for CoCl_2_ micro-injections (1 µl-25 mM) into the ventral hippocampus (coordinates from bregma: anterior/posterior − 3.3 mm, medial/lateral + 2.8 mm, dorsal/ventral − 4.0 mm). **g** Coronal sections of the ventral hippocampus of Saline and CoCl_2_ groups were stained with anti-EAAC1. Photomicrographs reveal EAAC1 expression in the DG (g), CA1 (i), and CA3 (k) regions of the ventral hippocampus in mice treated with saline (top panels) and CoCl_2_ (bottom panels). Scale bar, 100 µm; inset, enlarged areas. Scale bar, 25 µm. **h** Quantification analysis of EAAC1 immunoreactivity in **g**. **j** Quantification analysis of EAAC1 immunoreactivity in **i**. **l** Quantification analysis of EAAC1 immunoreactivity in **k**. Data on EAAC1 staining quantification are expressed as the means ± S.E.M.; n = 8; ***P < 0.001, *P < 0.05

We used CoCl_2_ to mimic hypoxia in SH-SY5Y cells. First, we examined whether CoCl_2_ altered the protein levels of EAAC1 in SH-SY5Y cells. We found that there was a dose-dependent increase in EAAC1 expression after 24 h of CoCl_2_ (50–500 µM) treatment (Fig. 1a). Quantification of the data demonstrated that CoCl_2_ significantly increased EAAC1 expression (CON, 1.04 ± 0.14; 50 μM CoCl_2_, 1.13 ± 0.29; 100 μM CoCl_2_, 1.71 ± 0.12; 150 μM CoCl_2_, 1.88 ± 0.18; 200 μM CoCl_2_, 2.58 ± 0.56; 300 μM CoCl_2_, 3.58 ± 0.56; 500 μM CoCl_2_, 5.87 ± 0.34; n = 8; ***P < 0.001; Fig. 1b). CoCl_2_ treatment significantly increased EAAC1 protein expression at each subsequent time point (0, 1, 3, 6, 12, 24, 36, and 48 h). EAAC1 protein expression was significantly increased after exposure to 100 μM CoCl_2_ for > 24 h compared with that of the controls (n = 6; *P < 0.05, ***P < 0.001; Fig. 1c and d). Next, we investigated whether EAAC1 overexpression affects the cellular cytotoxicity induced by CoCl_2_. Treatment with 100 µM CoCl_2_ for 36 h significantly induced cytotoxicity in both the Mock and EAAC1 transfection groups (Mock: CON, 25.85 ± 1.12; CoCl_2_, 57.43 ± 1.02; n = 4; ***P < 0.001, EAAC1-myc: CON, 23.03 ± 1.02; CoCl_2_, 48.88 ± 1.64; n = 4; ^###^*P* < 0.001; Fig. 1e). EAAC1 transfection reduced CoCl_2_-induced LDH release in SH-SY5Y cells (Mock: 57.43 ± 1.02; EAAC1-myc 48.88 ± 1.64; n = 4; ^++^*P* < 0.01, Fig. 1e).

We next analysed the expression of EAAC1 induced by CoCl_2_ microinjections in the VH in mice. EAAC1 protein levels were remarkably increased in the VH of the CoCl_2_ group (saline, 1.00 ± 0.15; CoCl_2_, 2.06 ± 0.09; t(14) = 5.680 in the DG; ***P < 0.001; saline, 1.00 ± 0.07; CoCl_2_, 1.40 ± 0.05; t(14) = 4.266 in the CA1; ***P < 0.001; saline, 1.00 ± 0.23; CoCl_2_, 1.52 ± 0.10; t(14) = 5.158 in the CA3; *P < 0.05; Fig. 1f-l).

### NRG1 alleviated CoCl_2_-induced upregulation of EAAC1 in SH-SY5Y cells and the hippocampus of mice

**Fig. 2:**
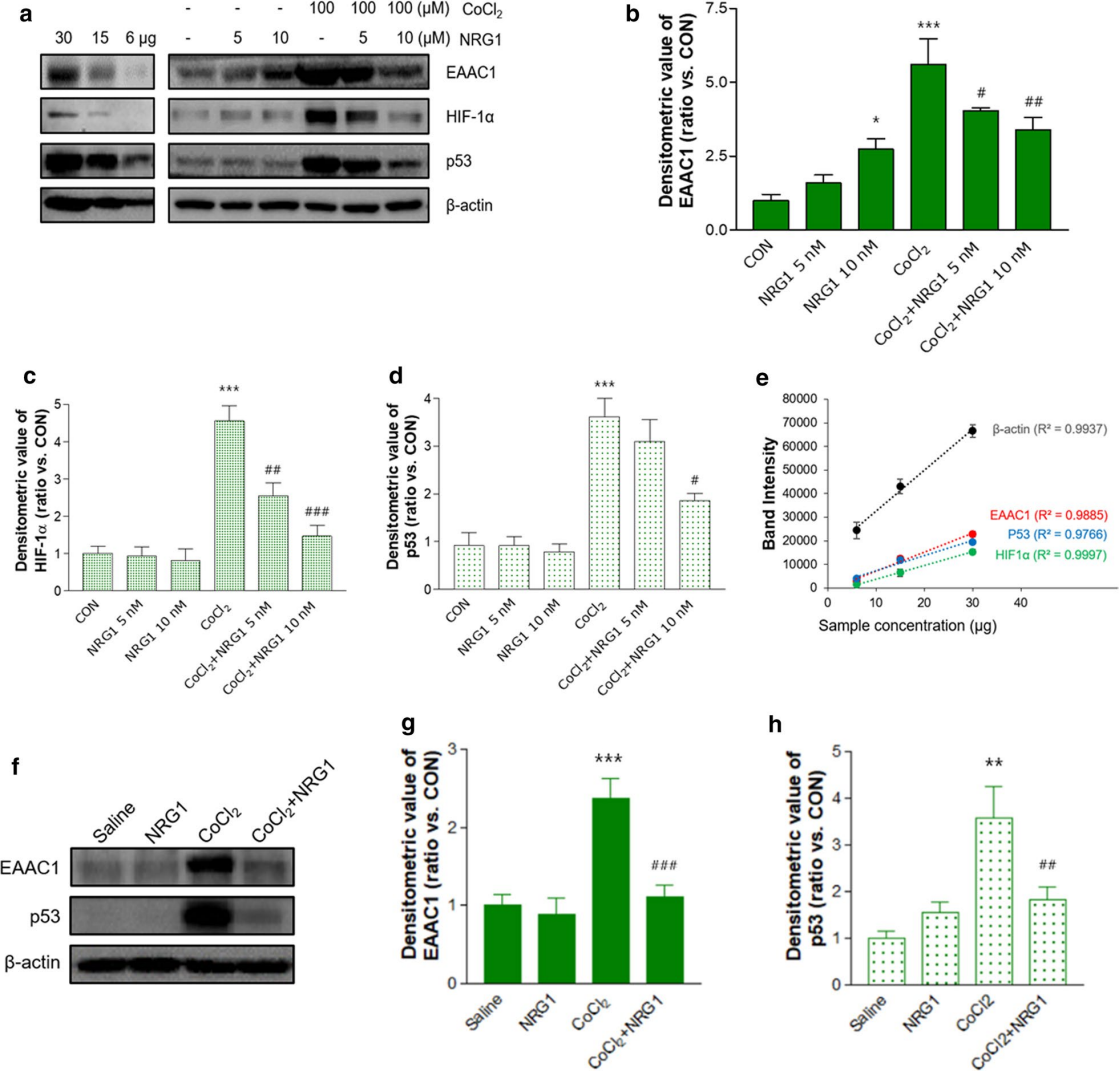
The effects of NRG1 on the CoCl_2_-induced protein levels of EAAC1. **a** Representative immunoblots of EAAC1, HIF-1α, and p53 in SH-SY5Y cells in the presence or absence of 5 nM or 10 nM NRG1 following treatment with 100 µM CoCl_2_ for 36 h are shown. Semiquantitative Western blot analysis of EAAC1 (upper panel), HIF-1α (middle panel), and p53 (lower panel) in SH-SY5Y cells. Cell lysates were loaded with a series of 50% and 20% dilutions from the cell extract. **b** Quantitative analysis of the data in a. Treatment with 100 µM CoCl_2_ significantly increased the expression of EAAC1. CoCl_2_-induced EAAC1 overexpression was attenuated by 5 nM or 10 nM NRG1 treatment. The densitometry values are shown as ratios relative to the values of the control group (n = 8; *P < 0.05, ***P < 0.001 versus the control group; ^#^*P* < 0.05, ^##^*P* < 0.01 versus the CoCl_2_ alone group). **c** Quantitative analysis of the data in a. CoCl_2_-induced HIF-1α accumulation was attenuated by 5 nM or 10 nM NRG1 treatment (n = 6; ***P < 0.001 versus the control group; ^##^*P* < 0.01, ^###^*P* < 0.001 versus the CoCl_2_ alone group). **d** Quantitative analysis of the data in a. CoCl_2_-induced p53 stability was attenuated by 10 nM NRG1 treatment (n = 6; ***P < 0.001 versus the control group; ^#^*P* < 0.05 versus the CoCl_2_ alone group). **e** Standard curve of semiquantitative Western blot data in a **f** Unilateral microinjection of CoCl_2_ into the VH increased the expression of EAAC1 and p53. NRG1 IP injection attenuates the overexpression of EAAC1 and p53. **g** Quantification of EAAC1 immunoreactivity in f (n = 8; ***P < 0.001 versus the control group; ^###^*P* < 0.001 versus the CoCl_2_ alone group). **h** Quantification of p53 immunoreactivity in f (n = 8; **P < 0.01 versus the control group; ^##^*P* < 0.01 versus the CoCl_2_ alone group)

To determine whether NRG1 affected the CoCl_2_-induced increase in EAAC1 expression, we pretreated cells with NRG1 (5 nM or 10 nM) for 15 min before CoCl_2_ administration. Treatment with 100 µM CoCl_2_ for 36 h significantly upregulated EAAC1 expression (CON, 0.99 ± 0.21; CoCl_2_, 5.61 ± 0.87; n = 8; ***P < 0.001; Fig. 2a and b). Treatment with 5 nM or 10 nM NRG attenuated the increase in EAAC1 expression induced by 100 µM CoCl_2_ (CoCl_2_, 5.61 ± 0.87; CoCl_2_ + 5 nM NRG1, 4.05 ± 0.09; CoCl_2_ + 10 nM NRG1, 3.39 ± 0.43; n = 8; ^#^*P* < 0.05, ^##^*P* < 0.01; Fig. 2a and b). As shown in Fig. 2a, c and d, treatment with 100 µM CoCl_2_ for 36 h significantly upregulated HIF-1α (CON, 1.01 ± 0.19; 100 µM CoCl_2_, 4.56 ± 0.41; n = 6; ***P < 0.001) and p53 (CON, 0.92 ± 0.27; 100 µM CoCl_2_, 3.62 ± 0.38; n = 6; ***P < 0.001) expression. Pretreatment with NRG1 for 36 h attenuated this increase in HIF-1α accumulation induced by 100 µM CoCl_2_ (CoCl_2_, 4.56 ± 0.41; CoCl_2_ + 5 nM NRG1, 2.55 ± 0.35; CoCl_2_ + 10 nM NRG1, 1.47 ± 0.28; n = 6; ^##^*P* < 0.01, ^###^*P* < 0.001; Fig. 2c). Moreover, pretreatment with 5 nM or 10 nM NRG1 for 36 h attenuated the increase in p53 stabilization induced by 100 µM CoCl_2_ (CoCl_2_, 3.62 ± 0.38; CoCl_2_ + 5 nM NRG1, 3.10 ± 0.46; CoCl_2_ + 10 nM NRG1, 1.85 ± 0.15; n = 6; ^#^*P* < 0.05; Fig. 2d). In addition, we confirmed these results based on a semiquantitative Western blot of EAAC1, HIF-1α, and p53 expression in SH-SY5Y cells (Fig. 2a and e).These results are consistent with those of our previous studies demonstrating the effects of NRG1 on HIF-1α or p53 [24]. To verify these results in vivo, we treated mice brains with vehicle or NRG1 (50 ng/kg, IP) for 3 days before CoCl_2_ microinjection into the VH. After CoCl_2_ microinjection, the mice continued receiving NRG1 for 2 days, and then the mice were sacrificed (Fig. 2f). Consistent with the in vitro results, NRG1 dramatically prevented the increase in EAAC1 (CON, 1.00 ± 0.14; NRG1, 0.89 ± 0.20; CoCl_2_, 2.38 ± 0.23; CoCl_2_ + NRG1, 1.11 ± 0.14; n = 5; ***P < 0.001, ^###^*P* < 0.001; Fig. 2g and h) and p53 expression (CON, 1.00 ± 0.13; NRG1, 1.57 ± 0.19; CoCl_2_, 3.59 ± 0.60; CoCl_2_ + NRG1, 1.82 ± 0.0.25; n = 5; **P < 0.01, ^##^*P* < 0.01; Fig. 2g and i) induced by CoCl_2_ microinjection in the hippocampus of the mouse brain.

### NRG1 inhibited CoCl_2_-induced increases in EAAC1, Tau, and phospho-Tau immunoreactivity

**Fig. 3:**
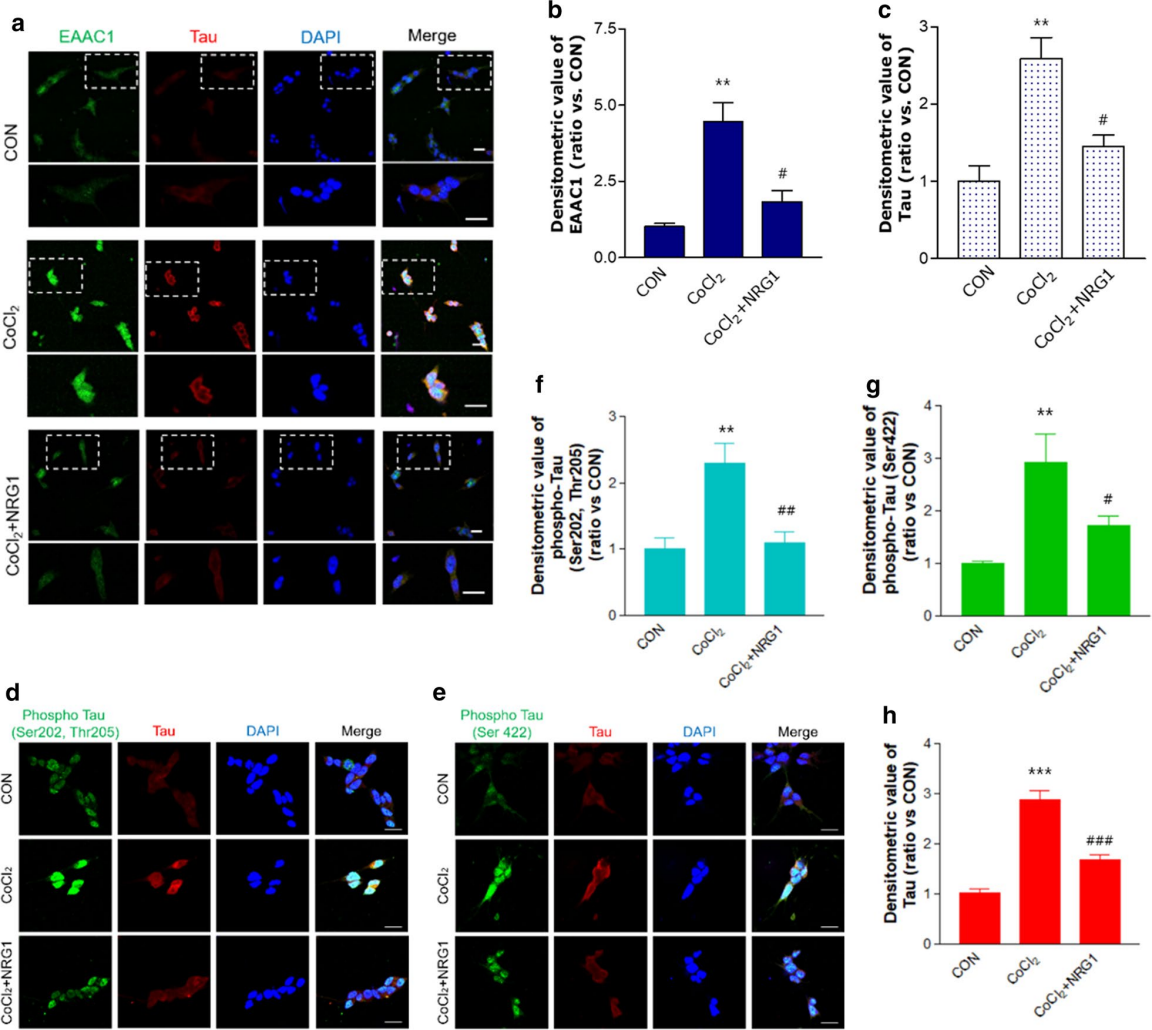
NRG1 attenuated the CoCl_2_-induced overexpression of EAAC1 and Tau in SH-SY5Y cells. **a** Immunofluorescence analysis with anti-EAAC1 and anti-Tau was performed 24 h after 100 µM CoCl_2_ treatment in the presence or absence of 10 nM NRG1 in SH-SY5Y cells. The cells were fixed and immunostained with anti-EAAC1 (green) and anti-Tau (red), while DAPI (blue) was used as a counterstain. The outlined image (upper) is enlarged (bottom). Scale bars, 20 µm. **b** Bar graph summarizing the data from neurons showing EAAC1 fluorescence (n = 8; **P < 0.01 versus the control group; ^#^*P* < 0.05 versus the CoCl_2_ alone group). **c** The fluorescence intensity of Tau was measured in each group (n = 8; **P < 0.01 versus the control group; ^#^*P* < 0.05 versus the CoCl_2_ alone group). **d** Expression of phospho-Tau (Ser202, Thr205) after 24 h of incubation under 100 µM CoCl_2_ treatment in the presence or absence of 10 nM NRG1 in SH-SY5Y cells as assessed by immunofluorescence. The cells were fixed and immunostained with anti-phospho-Tau (Ser202, Thr205) (green) and anti-Tau (red). Scale bars, 20 µm. **e** Immunofluorescence analysis with anti-phospho-Tau (Ser422) (green) and anti-Tau (red) was performed 24 h after 100 µM CoCl_2_ treatment in the presence or absence of 10 nM NRG1 in SH-SY5Y cells. Scale bars, 20 µm. **f** Bar graph summarizing the data from neurons showing phospho-Tau (Ser202, Thr205) fluorescence (n = 5; **P < 0.01 versus the control group; ^##^*P* < 0.01 versus the CoCl_2_ alone group). **g** The fluorescence intensity of phospho-Tau (ser422) was measured in each group (n = 6; **P < 0.01 versus the control group; ^#^*P* < 0.05 versus the CoCl_2_ alone group). **h** Quantification of data in **d** and **e.** The fluorescence intensity of Tau was measured in each group (n = 11; ***P < 0.001 versus the control group; ^###^*P* < 0.001 versus the CoCl_2_ alone group)

We examined the immunoreactivity of EAAC1 in SH-SY5Y cells using immunofluorescence staining. To measure the effects of NRG1 on SH-SY5Y cells, cells were pretreated for 15 min with 10 nM NRG1 and then treated with 100 µM CoCl_2_ (Fig. 3a). Treatment with 100 µM CoCl_2_ for 24 h significantly upregulated EAAC1 expression in comparison to that of the control group (CON, 1.02 ± 0.10; 100 µM CoCl_2_, 4.45 ± 0.64; n = 8; **P < 0.01; Fig. 3b). We also confirmed that the pretreatment of SH-SY5Y cells with 10 nM NRG1 for 24 h significantly attenuated EAAC1 overexpression (CoCl_2_, 4.45 ± 0.64; CoCl_2_ + 10 nM NRG1, 1.83 ± 0.37; n = 8; ^#^*P* < 0.05; Fig. 3b) compared with that of the control group. Interestingly, treatment with 100 µM CoCl_2_ for 24 h markedly increased the accumulation of Tau in comparison with that of the control group (CON, 1.00 ± 0.20; CoCl_2_, 2.58 ± 0.27; n = 8; **P < 0.01; Fig. 3c). Pretreatment with 10 nM NRG1 attenuated the CoCl_2_-induced increase in Tau expression (CoCl_2_, 2.58 ± 0.27; CoCl_2_ + NRG1, 1.45 ± 0.15; n = 8; ^#^*P* < 0.05; Fig. 3c). Furthermore, phospho-Tau (Ser202, Thr205) levels were increased in CoCl_2_-treated cells (CON, 1.00 ± 0.15; 100 µM CoCl_2_, 2.29 ± 0.28; n = 8; **P < 0.01; Fig. 3d and f), and pretreatment with 10 nM NRG1 attenuated the CoCl_2_-induced increase in phospho-Tau (Ser202, Thr205) expression (CoCl_2_, 2.29 ± 0.28; CoCl_2_ + NRG1, 1.10 ± 0.15; n = 5; ^##^*P* < 0.01; Fig. 3d and f). In addition, phospho-Tau (Ser422) levels were increased in CoCl_2_-treated cells (CON, 1.00 ± 0.04; 100 µM CoCl_2_, 2.92 ± 0.49; n = 6; **P < 0.01; Fig. 3e and g), and pretreatment with 10 nM NRG1 prevented the CoCl_2_-induced increase in phospho-Tau (Ser422) expression (CoCl_2_, 2.92 ± 0.49; CoCl_2_ + NRG1, 1.72 ± 0.17; n = 6; ^#^*P* < 0.05; Fig. 3e and g). We confirmed that 10 nM NRG1 pretreatment attenuated the CoCl_2_-induced increase in Tau expression (CON, 1.00 ± 0.13; CoCl_2_, 2.78 ± 0.28; CoCl_2_ + NRG1, 1.69 ± 0.08; n = 11; ***P < 0.001, ^###^*P* < 0.001; Fig. 3d, e, and h).

### NRG1 rescued CoCl_2_-induced ROS generation and the reduction in antioxidant enzymes in SH-SY5Y cells

**Fig. 4:**
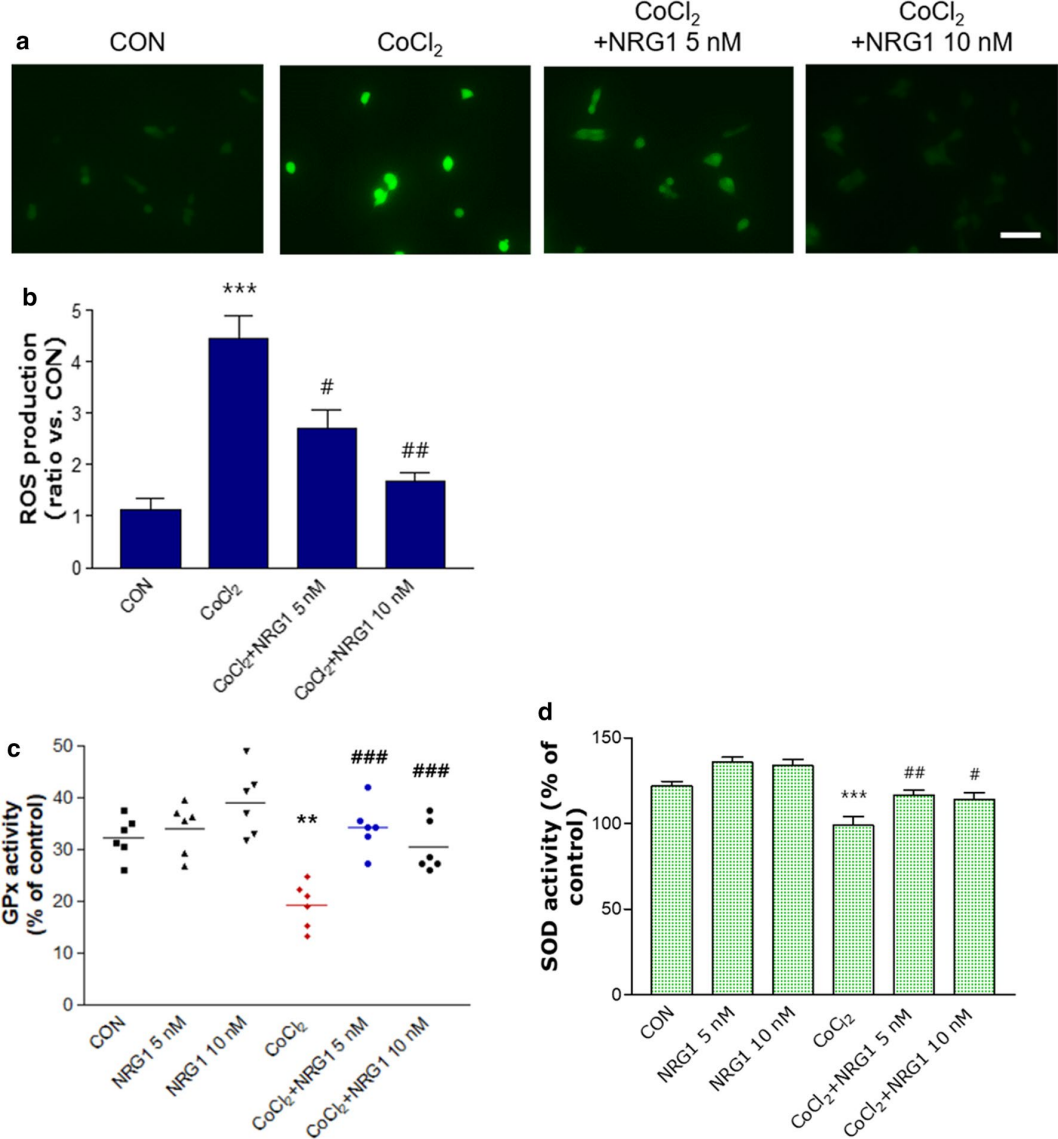
NRG1 reduced the increase in ROS and decreased oxidative stress-related enzyme activity induced by CoCl_2_. **a** After 24 h, intracellular ROS levels were measured by fluorescence microscopy using DCFH-DA dye that was administered to 100 µM CoCl_2_-treated SH-SY5Y cells that had been pretreated with NRG1 (5 or 10 nM) for 15 min. **b** Bar graph summarizing the data in a (n = 6; ***P < 0.001 versus the control group; ^#^*P* < 0.05, ^##^*P* < 0.01 versus the CoCl_2_ alone group). **c** SH-SY5Y cells were treated with 100 µM CoCl_2_ alone or with PBS or NRG1 (5 nM or 10 nM) for 36 h. GPx activity was measured using a GPx assay kit (Cayman Chemical Company, MI, USA) (n = 6; **P < 0.01 versus the control group; ^###^*P* < 0.001 versus the CoCl_2_ alone group). **d** After the SH-SY5Y cells were exposed to 100 µM CoCl_2_ in the presence or absence of NRG1 (5 nM or 10 nM) for 36 h, SOD activity was evaluated by measuring the inhibition of the reduction of tetrazolium salt by xanthine-xanthine oxidase according to the manufacturer’s instructions (Cayman Chemical Company, MI, USA). The SOD assay measured all three types of SOD (FeSOD, Cu/An, and Mn) (n = 8; ***P < 0.001 versus the control group; ^#^*P* < 0.05, ^##^*P* < 0.01 versus the CoCl_2_ alone group)

We tested the protective effect of NRG1 against CoCl_2_-induced ROS generation. We found that treatment with 100 µM CoCl_2_ for 24 h significantly increased ROS levels (CON, 1.13 ± 0.20; CoCl_2_, 4.46 ± 0.44; n = 6; ***P < 0.001; Fig. 4a and b) compared with the levels in the control group. However, pretreatment with 5 nM or 10 nM NRG1 significantly attenuated CoCl_2_-induced ROS generation (CoCl_2_, 4.46 ± 0.44; CoCl_2_ + 5 nM NRG1, 2.70 ± 0.37; CoCl_2_ + 10 nM NRG1, 1.67 ± 0.16; n = 6; ^#^*P* < 0.05, ^##^*P* < 0.01; Fig. 4a and b). To determine whether NRG1 affects the antioxidant defense system, we analyzed the activity of the antioxidant enzymes GPx and SOD. Treatment with 100 µM CoCl_2_ significantly reduced the activity of GPx (CON, 32.37 ± 1.63; CoCl_2_, 19.31 ± 1.77; n = 6; **P < 0.01; Fig. 4c) compared with that of the control group. Pretreatment with 5 nM or 10 nM NRG1 for 36 h attenuated the CoCl_2_-induced reduction in GPx activity (CoCl_2_, 19.31 ± 1.77; CoCl_2_ + 5 nM NRG1, 34.38 ± 1.94; CoCl_2_ + 10 nM NRG1, 30.46 ± 1.99; n = 6; ^###^*P* < 0.001; Fig. 4c). Moreover, after the cells were exposed to 100 µM CoCl_2_ in the presence or absence of NRG1 for 36 h, SOD activity was measured. We also demonstrated that after the cells were exposed to CoCl_2_ for 36 h, there were distinct decreases in SOD activity (CON, 121.78 ± 2.88; CoCl_2_, 98.91 ± 5.02; n = 8; ***P < 0.001; Fig. 4d). Moreover, pretreatment of cells with 5 nM or 10 nM NRG1 attenuated the CoCl_2_-induced decrease in SOD activity (CoCl_2_, 98.91 ± 5.02; CoCl_2_ + 5 nM NRG1, 116.74 ± 2.51; CoCl_2_ + 10 nM NRG1, 114.189 ± 3.76; n = 8; ^#^*P* < 0.05, ^##^*P* < 0.01; Fig. 4d).

### NRG1 treatment reduced superoxide generation induced by microinjection of CoCl_2_ into the VH of mice.

**Fig. 5:**
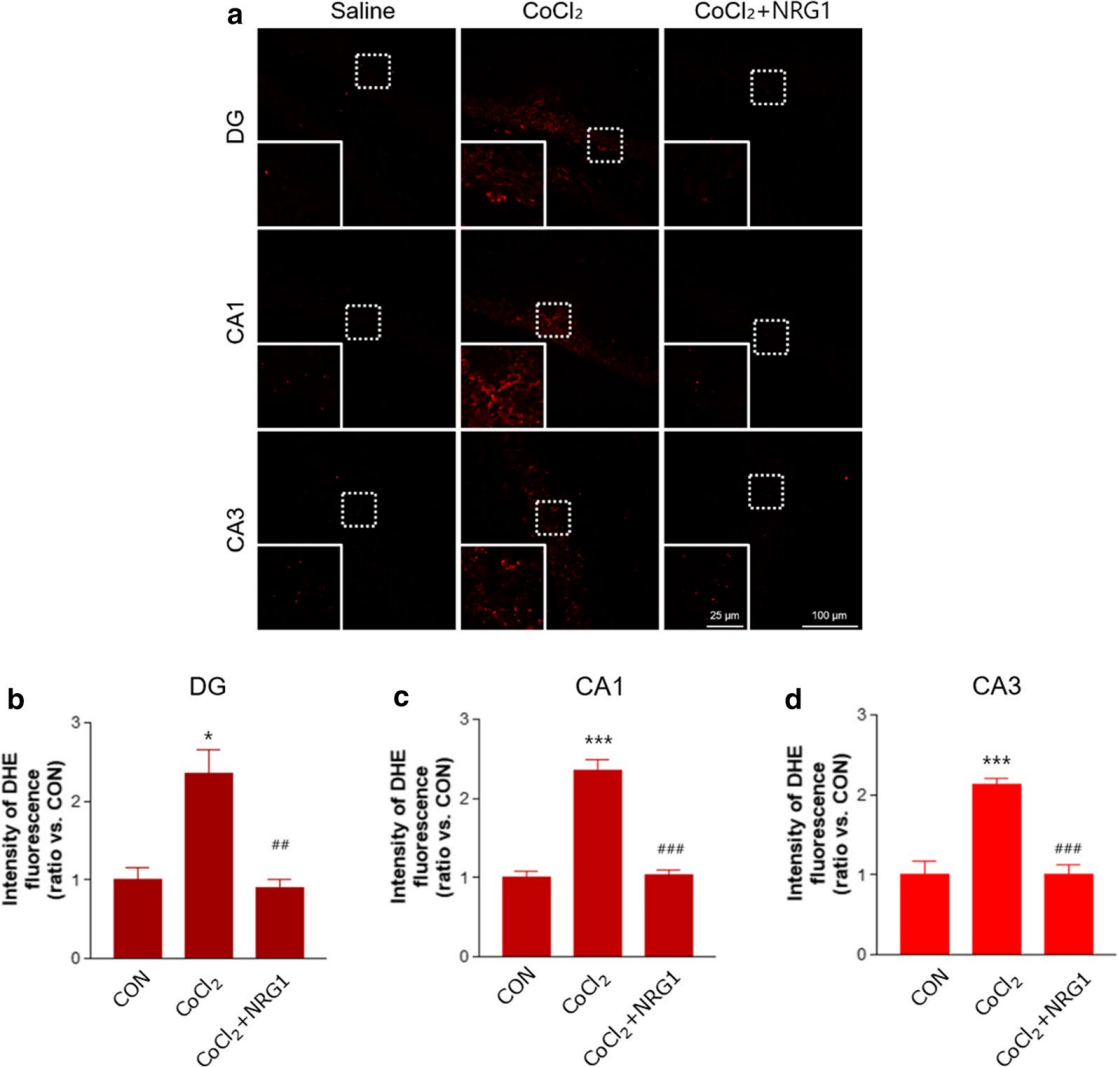
NRG1 treatment reduced superoxide generation induced by microinjection of CoCl_2_ into the VH of mice. **a** Photomicrographs reveal the generation of superoxide in the DG (top panels), CA1 (middle panels), and CA3 (bottom panels) regions of the VH in the Saline, CoCl_2,_ and CoCl_2_ + NRG1 groups. Increased superoxide generation of VH induced by microinjection with CoCl_2_ (1 µl-25 mM) was measured by fluorescence microscopy using the DHE dye. The mice were treated with NRG1 (50 ng/kg) for 5 days. Scale bar, 100 µm; *inset*, enlarged areas. Scale bar, 25 µm. **b** Bar graph summarizing the data in a (DG) (n = 8; *P < 0.05 versus the saline group; ^##^*P* < 0.01 versus the CoCl_2_ alone group). **c** Bar graph summarizing the data in a (CA1) (n = 8; ***P < 0.001 versus the saline group; ^###^*P* < 0.001 versus the CoCl_2_ alone group). **d** Bar graph summarizing the data in a (CA2) (n = 8; ***P < 0.001 versus the saline group; ^###^*P* < 0.001 versus the CoCl_2_ alone group)

To further demonstrate that NRG1 protects against ROS generation in vivo, we determined the amount of hippocampal superoxide content as a major ROS form by DHE staining. We investigated the generation of superoxide in the DG (top panels), CA1 (middle panels), and CA3 (bottom panels) regions of the VH by microinjection of CoCl_2_ (DG: saline, 1.00 ± 0.13; CoCl_2_, 2.36 ± 0.27; n = 8; *P < 0.05; CA1: saline, 1.00 ± 0.08; CoCl_2_, 2.36 ± 0.14; n = 8; ***P < 0.001; CA3: saline, 1.00 ± 0.15; CoCl_2_, 2.13 ± 0.07; n = 8; ***P < 0.001; Fig. 5a-d). NRG1 injection in mice of micro-injected CoCl_2_ alleviated the increase in superoxide generation in the DG, CA1, and CA3 (DG: CoCl_2_, 2.36 ± 0.27; CoCl_2_ + NRG1, 0.90 ± 0.09; n = 8; ^##^*P* < 0.01; CA1: CoCl_2_, 2.36 ± 0.14; CoCl_2_ + NRG1, 1.04 ± 0.05; n = 8; ^###^*P* < 0.001; CA3: CoCl_2_, 2.13 ± 0.07; CoCl_2_ + NRG1, 1.01 ± 0.11; n = 8; ^###^*P* < 0.001; Fig. 5a-d).

### NRG1 rescued CoCl_2_-induced apoptosis and cell death

**Fig. 6:**
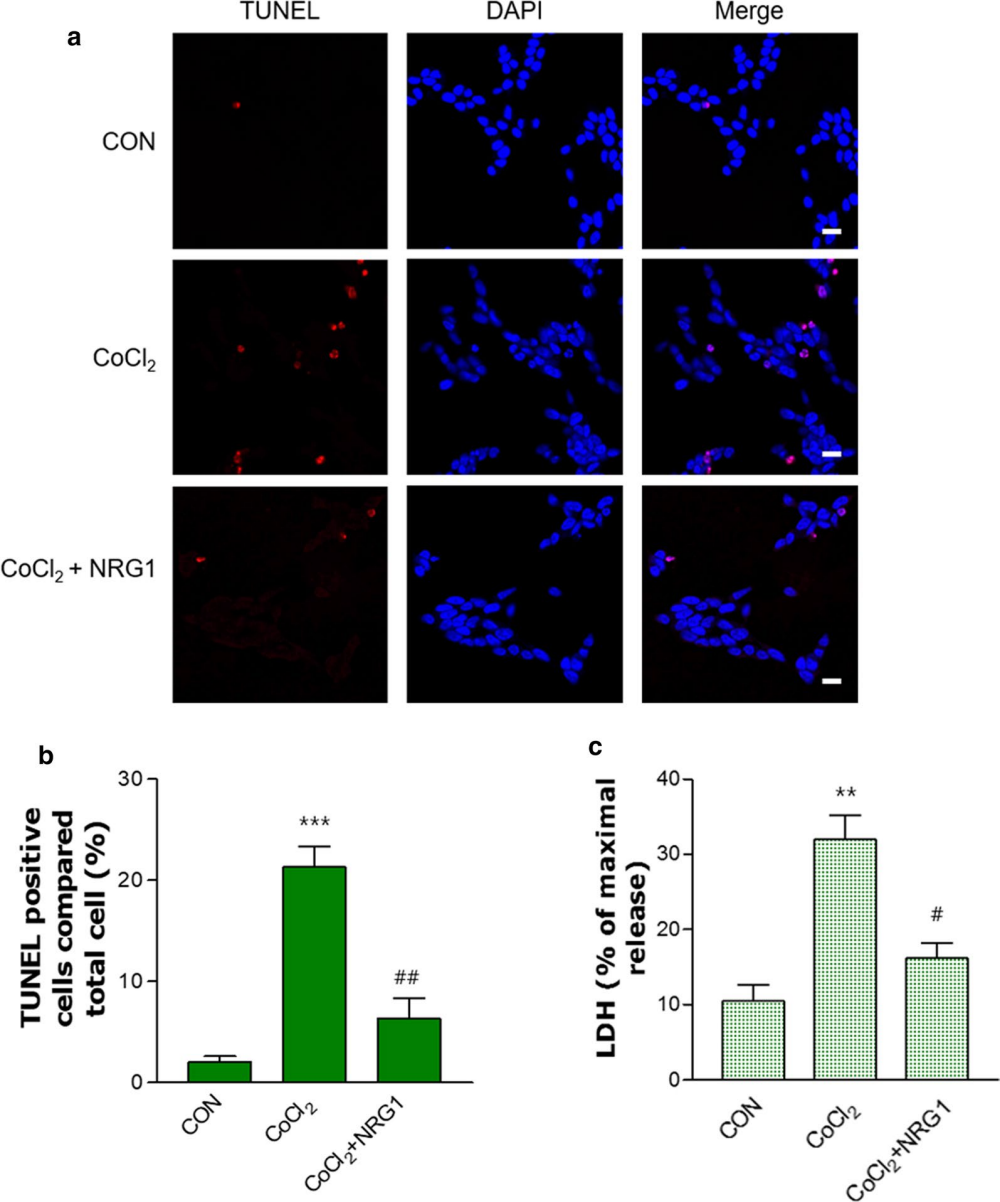
NRG1 attenuated CoCl_2_-induced apoptosis and cell death in SH-SY5Y cells. **a** TUNEL staining (red) showing the amount of apoptosis that occurred in cells treated with 100 µM CoCl_2_ and either PBS or 10 nM NRG1 for 24 h. DAPI (blue) was used as a counterstain. Scale bars, 20 µm. **b** Apoptotic cells are expressed as the percentage of TUNEL-positive cells relative to the number of DAPI-stained cells (n = 6; ***P < 0.001 versus the control group; ^##^*P* < 0.01 versus the CoCl_2_ alone group). **c** NRG1 (10 nM) attenuated cell death induced by 100 µM CoCl_2_ in SH-SY5Y cells. After 36 h, the degree of cell death was measured by LDH activity in the medium (n = 6; **P < 0.01 versus the control group; ^#^*P* < 0.05 versus the CoCl_2_ alone group)

We examined whether NRG1 affects CoCl_2_-induced apoptosis in SH-SY5Y cells. To detect apoptotic nuclei in SH-SY5Y cells, we used TUNEL staining. Treatment with 100 µM CoCl_2_ significantly increased the proportion of apoptotic nuclei (CON, 2.00 ± 0.58; CoCl_2_, 21.33 ± 2.03; n = 6; ***P < 0.001; Fig. 6a and b) compared with that of the control group. Pretreatment with 10 nM NRG1 for 24 h reduced the number of CoCl_2_-induced TUNEL-positive cells (CoCl_2_, 21.33 ± 2.03; CoCl_2_ + 10 nM NRG1, 6.33 ± 2.03; n = 6; ^##^*P* < 0.01, Fig. 6a and b).

Next, we examined CoCl_2_-induced cytotoxicity in SH-SY5Y cells. The cells were incubated with 10 nM NRG1 and then exposed to 100 μM CoCl_2_ for 36 h (CON, 10.48 ± 2.10; CoCl_2_, 31.97 ± 3.21; CoCl_2_ + 10 nM NRG1, 16.18 ± 2.05; n = 6; **P < 0.01, ^#^*P* < 0.05; Fig. 6c).

### Effects of NRG1 on CoCl_2_-induced changes in apoptotic or antiapoptotic proteins

**Fig. 7:**
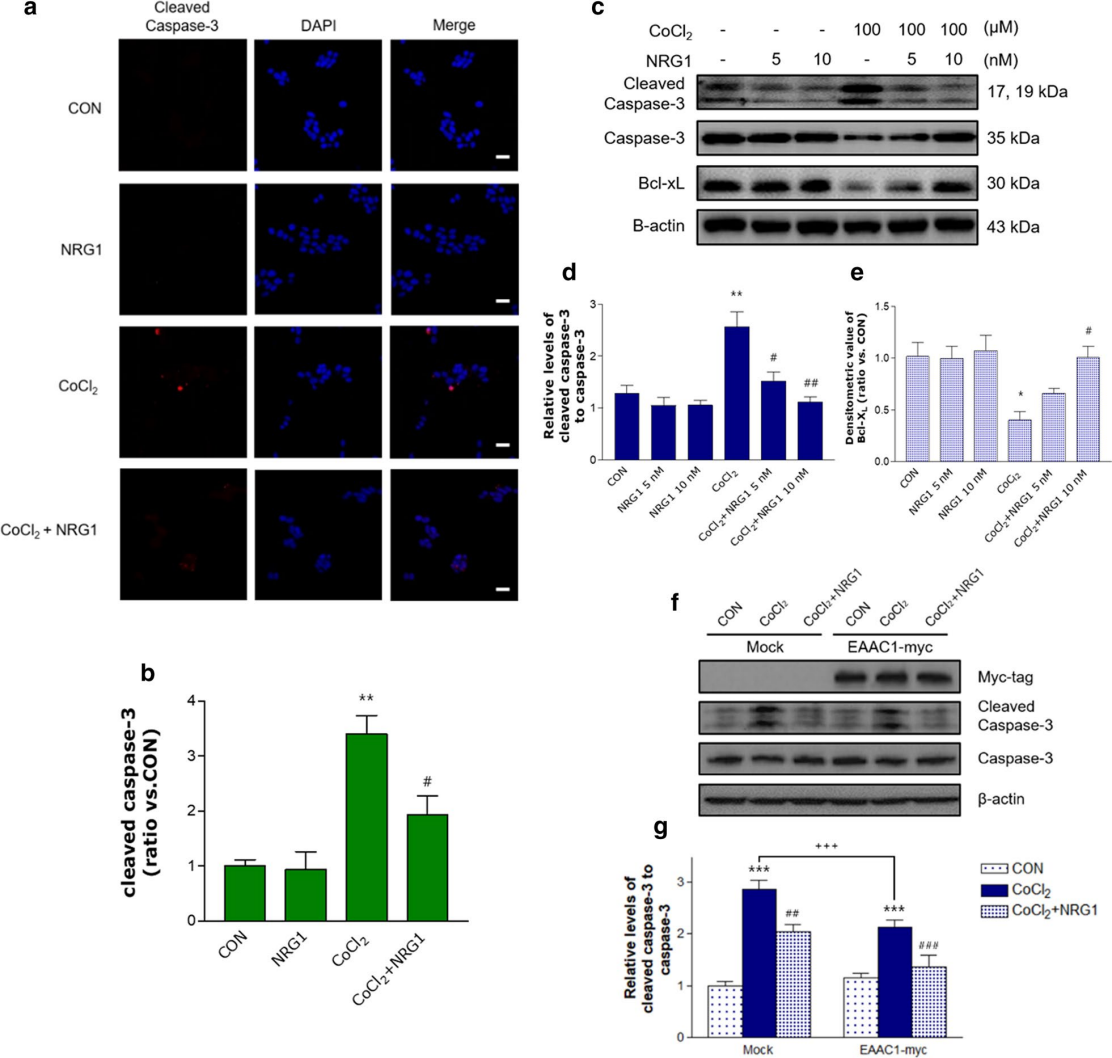
The effects of NRG1 on the CoCl_2_-induced protein levels of cleaved caspase-3 and Bcl-X_L_. **a** Representative immunofluorescence image of cells after treatment with 100 µM CoCl_2_ in the presence or absence of 10 nM NRG1 for 24 h. Cells were fixed and immunostained with anti-cleaved caspase-3 (red) and counterstained with DAPI (blue). Cleaved caspase-3 staining was significantly higher in CoCl_2_-only treated cells than in control cells. NRG1 protected against CoCl_2_-induced expression of cleaved caspase-3. **b** The ratio of cleaved caspase-3-positive cells to the total number of cells (n = 6; **P < 0.01 versus the control group; ^#^*P* < 0.05 versus the CoCl_2_ alone group). Scale bars, 20 μm. **c** Protein expression of Bcl-X_L_, cleaved caspase-3, caspase-3 and β-actin was analyzed by western blotting. Representative immunoblots showing SH-SY5Y cells treated with 100 µM CoCl_2_ and either PBS or NRG1 (5 nM or 10 nM) for 36 h. **d** Quantitative analysis of the data in a. Treatment with CoCl_2_ significantly increased the expression of cleaved caspase-3. NRG1 attenuated the increase in cleaved caspase-3 expression, as shown by the densitometric values (n = 6; **P < 0.01 versus the control group; ^#^*P* < 0.05, ^# #^*P* < 0.01 versus the CoCl_2_ alone group). e Quantitative analysis of the data in a. Treatment with CoCl_2_ significantly decreased the expression of Bcl-X_L_ in SH-SY5Y cells. NRG1 inhibited the reduction in Bcl-X_L_ expression, as shown by the densitometric values (n = 6; *P < 0.05 versus the control group; ^#^*P* < 0.05 versus the CoCl_2_ alone group). **f** Protein expression of myc-tag, cleaved caspase-3, caspase-3, and β-actin was analysed after EAAC1-myc transfection. **g** Representative immunoblots of SH-SY5Y cells treated with 100 µM CoCl_2_ and either PBS or NRG1 (5 nM) for 36 h. Quantitative analysis of the data in f (Mock: n = 6; ***P < 0.001 versus the control group; ^##^*P* < 0.01 versus the CoCl_2_ alone group: EAAC1-myc: n = 6; ***P < 0.001 versus the control group; ^###^*P* < 0.001 versus the CoCl_2_ alone group). EAAC1 transfection reduced CoCl_2_-induced cleaved caspase-3 (n = 6; ^+++^*P* < 0.001)

We next investigated whether caspase-3 cleavage is increased by CoCl_2_. SH-SY5Y cells were treated with 100 µM CoCl_2_ for 24 h before fixation and immunofluorescence detection of cleaved caspase-3. We found that CoCl_2_ increased the cleavage of caspase-3, and quantitative analysis showed that the number of cleaved caspase-3-positive cells was increased (CON, 1.00 ± 0.51; CoCl_2_, 3.40 ± 0.34; n = 6; **P < 0.01; Fig. 7a and b). Furthermore, pretreatment with 10 nM NRG1 for 24 h rescued the CoCl_2_-induced increase in the number of cleaved caspase-3-positive cells (CoCl_2_, 3.40 ± 0.34; CoCl_2_ + 10 nM NRG1, 1.93 ± 0.35; n = 6; ^#^*P* < 0.05, Fig. 7a and b). To determine whether NRG1 regulates CoCl_2_-induced caspase-3 cleavage, we performed western blotting. We observed that the level of cleaved caspase-3 (17 and 19 kD) was significantly increased after CoCl_2_ treatment (CON, 1.28 ± 0.16; CoCl_2_, 2.56 ± 0.29; n = 6; **P < 0.01; Fig. 7c and d). NRG1 attenuated the CoCl_2_-induced increase in cleaved caspase-3 (CoCl_2_, 2.56 ± 0.29; CoCl_2_ + 5 nM NRG1, 1.52 ± 0.17; CoCl_2_ + 10 nM NRG1, 1.13 ± 0.10; n = 6; ^#^*P* < 0.05, ^##^*P* < 0.01; Fig. 7c and d). Furthermore, the expression of Bcl-X_L_ (an antiapoptotic protein) was decreased in CoCl_2_-induced cells (CON, 1.02 ± 0.14; CoCl_2_, 0.4 ± 0.08; n = 6; *P < 0.05; Fig. 7c and e). NRG1 protected against the CoCl_2_-induced reduction in Bcl-xL protein expression (CoCl_2_, 0.4 ± 0.08; CoCl_2_ + 5 nM NRG1, 0.66 ± 0.05; CoCl_2_ + 10 nM NRG1, 1.01 ± 0.11; n = 6; ^#^*P* < 0.05; Fig. 7c and e). These results suggest that NRG1 may have a protective role under hypoxic conditions by regulating apoptosis. In addition, we confirmed the effect of NRG1 on the upregulation of EAAC1 by transient transfection in CoCl_2_-induced apoptosis. EAAC1 transfection reduced CoCl_2_-induced caspase-3 cleavage (Mock: 2.78 ± 0.13; EAAC1-myc: 2.13 ± 0.14; n = 6; ^+++^*P* < 0.01; Fig. 7f and g). NRG1 attenuated CoCl_2_-induced increases in cleaved caspase-3 in both Mock (CON, 1.00 ± 0.08; CoCl_2_, 2.78 ± 0.13; CoCl_2_ + 5 nM NRG1, 2.18 ± 0.11; n = 6; ***P < 0.001, ^##^*P* < 0.01; Fig. 7f and g) and EAAC1-myc (CON, 1.14 ± 0.09; CoCl_2_, 2.13 ± 0.14; CoCl_2_ + 5 nM NRG1, 1.28 ± 0.20; n = 6; ***P < 0.001, ^###^*P* < 0.001; Fig. 7f and g).

## Discussion

In the present study, we assessed the effects and mechanisms of NRG1 on CoCl_2_-induced oxidative stress in SH-SY5Y cells and the hippocampus of mice. First, we demonstrated that CoCl_2_ dramatically increased EAAC1 protein expression in SH-SY5Y cells. We also confirmed the increased EAAC1 expression by CoCl_2_ microinjection in the VH in mice. EAAT1 and EAAT2 are mainly expressed in glial cells [27–29], whereas EAAT3 is exclusively expressed in neurons [30–33]. The EAAC1 protein is abundantly expressed in the hippocampus, cerebellum, and midbrain areas [31]. In general, EAAC1 activity is considered to be the main mechanism responsible for glutamatergic transmission [2], and EAAC1 also transports cysteine into neurons [34, 35]. Modulation of EAAC1 activity correlates with neuronal GSH levels [7] and the rate-limiting substrate for neuronal synthesis of GSH [36].

EAAC1 may be the major contributor to GSH synthesis [5] in neurons. Interestingly, Rossi et al. reported that glutamate release is largely mediated by reversed activity of the neuronal glutamate transporter in severe brain ischemia. The glutamate transporter plays a key role in generating anoxic depolarization in hippocampal neurons [37]. These results suggest that the abnormal activity abolished information processing in the CNS within minutes of ischemia EAAC1-deficient mice showed that the delayed anoxic depolarization [38], overexpression of EAAC1 could contribute to the reversed activity in neurons. *SLC1A1* encodes EAAC1, a *SLC1A1* polymorphism highly replicated in obsessive–compulsive disorder studies that is associated with increased transcript levels in human brain tissue [39, 40]. Mice with EAAC1 overexpression displayed increased anxiety-like and repetitive behaviours and synaptic alterations [41]. Even if our data demonstrate that the transient transfection of EAAC1-myc reduced CoCl_2_-induced cell death and oxidative stress in SH-SY5Y cells, the abnormal overexpression of EAAC1 by chronic hypoxic stress might alter synaptic function and neuronal circuits in animal models.

Hypoxic conditions have been extensively studied for their potential to regulate glutamate transporters, as this putative regulation could have important consequences for brain pathologies. A previous study reported that chronic hypoxia upregulates EAAC1 expression in PC12 cells [42]. CoCl_2_ was reported to be a widely used hypoxia mimetic in a large variety of cells and is known to both inhibit prolyl hydroxylases, leading to HIF-1α stabilization, and induce ROS formation under normoxic conditions [43, 44]. In addition, direct CoCl_2_ brain microinjection provides a valuable animal model to develop focal ischemia in selected brain regions to study their functional consequences and potential pharmacological therapies.

Furthermore, we examined the effect of NRG1 on CoCl_2_-induced EAAC1 and hypoxia-related protein. Several lines of evidence collectively suggest that NRG1 plays a neuroprotective role in the brain against neurotoxic substances related to apoptosis and oxidative damage in neurons [45–48]. In this study, we showed that NRG1 could prevent CoCl_2_-induced upregulation of EAAC1 levels in SH-SY5Y cells and the hippocampus of brain. We also confirmed that NRG1 could attenuate the CoCl_2_-induced accumulation of HIF-1α and p53 [24]. Immunofluorescence analysis also showed that NRG1 significantly inhibited CoCl_2_-induced overexpression of EAAC1 in SH-SY5Y cells. Tau protein is a soluble microtubule-associated protein that is abundant in neurons and plays a role in neurite outgrowth and axonal transport [49, 50]. Additionally, the level of Tau and phospho-Tau increased in cells after CoCl_2_ treatment, suggesting that hypoxia or oxidative stress can lead to alterations in cell structure. Previously, there was a report showing that hypoxia promoted the phosphorylation and total expression of tau protein [42, 51]. Additional evidence suggests that hypoxic and ischaemic brain damage in humans and animals may contribute to tau protein dysfunction, which is proposed as a risk factor for developing Alzheimer’s disease (AD) [52]. The model generated using the hypoxia-mimicking agent CoCl_2_ excluded environmental and vascular factors; thus, it could be useful to investigate the correlation between cellular hypoxia and AD. Moreover, we found that NRG1 prevented the CoCl_2_-induced upregulation of EAAC1, Tau and phospho-Tau.

Next, we examined whether NRG1 protects against CoCl_2_-induced ROS generation. Numerous studies have suggested that hypoxia induces increased production of ROS in the brain [53–55]. When we treated the cells with CoCl_2_, ROS levels were increased. According to our results, NRG1 attenuated the CoCl_2_-induced generation of ROS in SH-SY5Y cells. There is a balance between the generation of ROS and their clearance by antioxidant networks, mainly by GPx, SOD, and catalase under physiological conditions [56, 57]. In the present study, CoCl_2_ reduced the activity of GPx and SOD in SH-SY5Y cells. We found that NRG1 had a protective effect on the CoCl_2_-induced reduction in GPx and SOD enzymatic activity. Furthermore, we confirmed that NRG1 reduced superoxide generation induced by microinjection of CoCl_2_ into the VH of brain. ROS is a powerful initiator of apoptosis, which also contributes to hypoxia-mediated neuronal cell death [58]. We also found that NRG1 significantly reduced CoCl_2_-induced apoptosis and cell death in SH-SY5Y cells.

In the intrinsic pathway, ROS induce mitochondria-dependent apoptosis. This process can be modulated by the release of cytochrome c and the downstream activation of caspases. We next focused on whether NRG1 could protect SH-SY5Y cells against the activation of caspase-3 after CoCl_2_ treatment. Our results verified that NRG1 significantly reduced the expression of cleaved caspase-3, which may have prevented hypoxia-induced apoptosis and cell death in SH-SY5Y cells. Immunoblot analysis also confirmed the effect of NRG1 on the CoCl_2_-induced activation of caspase-3. Bcl-2 family members act as critical regulators of the intrinsic apoptotic pathway. The antiapoptotic Bcl-2 family protein Bcl-X_L_ predominantly localizes to the outer mitochondrial membrane, whereas other members indirectly interact with mitochondria [59]. We further confirmed that NRG1 inhibited the CoCl_2_-induced reduction in Bcl-xL expression. Taken together, our data suggest that NRG1 protects against CoCl_2_-induced overexpression of EAAC1.

Pretreatment with NRG1 could activate these cellular defense mechanisms to mimic hypoxic preconditioning. NRG1 exerts its biological effects by activating a family of ErbB tyrosine kinase receptors. NRG1 can trigger signaling pathways such as Raf-MEK-ERK and PI3K-Akt-S6K. Further study is needed to clarify the underlying pathway associated with NRG1 in these effects.

## Conclusion

Our study suggests that CoCl_2_ significantly increases EAAC1 expression in SH-SY5Y cells and the hippocampus of mice. NRG1 attenuates the CoCl_2_-induced overexpression of EAAC1 and reduces CoCl_2_-induced oxidative stress and apoptotic signaling. NRG1 potentially plays a protective role in hypoxia through the inhibition of oxidative stress and maintains normal EAAC1 expression levels.

These results may show a new path toward understanding the pathogenesis and treatment of hypoxia and oxidative stress-related neurological diseases.

## Data Availability

Please contact author for data requests.

## Abbreviations

EAAC1: Excitatory amino acid carrier 1 (also referred to as EAAT3)
EAAT: Excitatory amino acid transporter
CNS: Central nervous system
GSH: Glutathione
HIF-1: Hypoxia inducible factor-1
OGD: Oxygen–glucose deprivation
NRG1: Neuregulin 1
CoCl_2_: Cobalt chloride
ROS: Reactive oxygen species
LDH: Lactate Dehydrogenase
TUNEL: Terminal deoxynucleotidyl transferase dUTP nick end labeling
GPx: Glutathione peroxidase
SOD: Superoxide dismutase
DHE: Dihydroethidium
VH: Ventral hippocampus
